# 基于琼脂糖凝胶电泳的二进制加密综合实验

**DOI:** 10.3724/SP.J.1123.2025.10029

**Published:** 2026-04-08

**Authors:** Jinwei DUAN, Lei MA, Qian ZHAO, Qianqian WU, Boyu XIN, Jiahua YANG, Yao LI, Qizhao WANG

**Affiliations:** 1.长安大学理学院，陕西 西安 710064; 1. College of Sciences，Chang’an University，Xi’an 710064，China; 2.陕西维世诺生物科技有限公司，陕西 西安 710304; 2. Shaanxi Weishinuo Biotechnology Co. Ltd. ，Xi’an 710304，China; 3.长安大学材料科学与工程学院，陕西 西安 710064; 3. School of Materials Science and Engineering，Chang’an University，Xi’an 710064，China; 4.长安大学地球科学与资源学院，陕西 西安 710054; 4. School of Earth Sciences and Resources，Chang’an University，Xi’an 710054，China; 5.长安大学水利与环境学院，陕西 西安 710054; 5. School of Water and Environment，Chang’an University，Xi’an 710054，China

**Keywords:** 脱氧核糖核酸纳米开关, 琼脂糖凝胶电泳, 核糖核酸调控保护, 核糖核酸酶A, 信息加密与解密, deoxyribonucleic acid nanoswitch （DNA nanoswitch）, agarose gel electrophoresis, ribonucleic acid-regulated protection, ribonuclease A, information encryption and decryption

## Abstract

本实验基于一个4位（4-bit）脱氧核糖核酸（DNA）纳米开关，通过特定DNA单链触发“线性-环状”构象转换，利用二者在凝胶电泳中的迁移差异实现数字的二进制编码。当不同构象开关组合时，这些组合可表示不同信息。本实验受有机化学“保护-脱保护”策略启发，引入核糖核酸（RNA）保护链以阻止环状结构形成，构建了信息加密系统。该系统利用核糖核酸酶A特异性酶切脱保护，恢复信息读取，构建了RNA调控的加密系统。本实验通过整合DNA纳米技术、二进制编码与化学保护策略，使琼脂糖凝胶电泳贯穿整个实验，实现了从分子构建到信息读写的全程可视化。这不仅使学生掌握了凝胶电泳操作，深化了对电泳分离机制与分子构效关系的理解，而且通过接触分子信息编码、DNA纳米技术等前沿领域，有效激发了学生的创新思维与跨学科问题的解决能力。

## Abstract (en)

This experiment is based on a 4-bit deoxyribonucleic acid （DNA） nanoswitch， in which specific DNA single strands trigger a “linear-to-circular” conformational transition. By leveraging the migration differences between the two conformations in gel electrophoresis， digital binary encoding is achieved. Different combinations of conformational switches can represent distinct information. Inspired by the “protection-deprotection” strategy in organic chemistry， an ribonucleic acid （RNA） protection strand is introduced to prevent the formation of the circular structure， thereby establishing an information encryption system. The system utilizes ribonuclease A （RNase A） for specific enzymatic cleavage to remove the protection， restoring information readout and establishing an RNA-regulated encryption system. By integrating DNA nanotechnology， binary encoding， and chemical protection strategies， agarose gel electrophoresis is applied throughout the entire experimental process， enabling full visualization from molecular construction to information read-write. This approach not only helps students master gel electrophoresis techniques and deepen their understanding of electrophoretic separation mechanisms and structure-activity relationships at the molecular level， but also exposes them to cutting‑edge fields such as molecular information encoding and DNA nanotechnology， effectively stimulating innovative thinking and interdisciplinary problem-solving skills.

将前沿科学成果融入本科理论和实验教学已成为新工科背景下的重要育人路径^［1-4］^。这种方式不仅能打破传统课程的知识壁垒，而且通过模块化设计可适配教学的可操作性，并以其前沿性特征有效激发学生创新思维^［5，6］^。

然而，当前大学化学课程如《无机化学》《物理化学》等虽已涵盖电泳原理并开设相关实验，但学生的认知多停留于原理和操作层面，对电泳在生物分子分离及表征等领域的系统认知仍显不足。近年来，科学家开发了识别生肉物种来源的等电点条形码^［7，8］^、基于实时聚丙烯酰胺凝胶电泳检测多糖的糖类图谱仪^［9］^，以及基于电泳图构建的鉴别不同重构治疗性单克隆抗体方法^［10］^等。由此可见，学生的认知断层与凝胶电泳的应用潜力共同凸显了创新设计综合凝胶电泳实验的必要性^［11，12］^。

脱氧核糖核酸（DNA）纳米技术凭借碱基互补配对的精准可控性，已在信息加密等领域展现出巨大的应用潜力^［13-16］^。琼脂糖凝胶电泳因操作简便、结果可视化等优点，成为该领域的通用表征技术，在实验教学中具有很高的教学价值。最近，长安大学段金伟课题组^［17，18］^、西北大学陈方方课题组^［19］^尝试将DNA纳米技术和前沿科研成果引入本科实验教学中，取得了很好的教学成效。近年来，Halvorsen等^［20，21］^报道了一个全新的纳米级的定性定量分析实验平台-DNA纳米开关；与传统凝胶电泳依靠相对分子质量差异分离不同，DNA纳米开关利用构型差异实现区分。这一特性为在本科实验中帮助学生深入理解分子构型与电泳迁移速率之间的关系提供了绝佳载体。

基于DNA纳米开关，Chandrasekaran等^［22］^利用线性与环状DNA纳米开关在电泳迁移率上的差异，设计了具有不同分子直径的DNA纳米开关，并结合核酸核糖酶H（ribonuclease H， RNase H）的精准调控，成功实现了分子级字母信息编码。与传统电子加密技术相比，DNA加密技术凭借其独特的分子自组装特性和酶响应机制，在信息安全性、存储密度等方面展现出巨大潜能。这自然引出一个值得探索的问题：如何利用琼脂糖凝胶电泳的迁移特性构建二进制信息编码方案，并进一步借助DNA纳米开关的构象转换机制实现该信息的加密与解密？

为此，本实验以DNA纳米开关为基础，将二进制信息表达与有机化学中的“保护-脱保护”策略等相融合，设计了一种由核糖核酸（RNA）调控的二进制加密创新机制。通过不同DNA纳米开关组合实现二进制信息的高效存储，利用RNA与检测链的配对形成位阻，实现信息加密，借助核酸核糖酶A（ribonuclease A，RNase A）降解RNA链脱保护实现解密。最后，通过琼脂糖凝胶电泳条带差异实现信息可视化读取。整个实验中，琼脂糖凝胶电泳技术贯穿了DNA纳米开关制备及表征、机理验证、非特异性酶攻击测试以及数据加密解密等环节。此外，我们还对该综合实验实施了模块化设计。该实验不仅能帮助学生牢牢掌握凝胶电泳技术的原理和操作，深入理解其在DNA纳米开关分子构效关系解析中的应用，而且通过“分子编程”理念使学生直观接触DNA纳米技术和分子加密等前沿领域，从而有效促进跨学科思维的培养。

## 1 实验原理

### 1.1 电泳原理

在外电场的作用下，带有电荷的溶胶粒子作定向迁移，称为电泳^［23］^。影响电泳的因素有相对分子质量、粒子大小与形状、表面电荷数目、溶剂中电解质的种类与离子强度、pH、温度及所加电压等。电泳类型多样，生物化学中常用凝胶电泳，如琼脂糖凝胶电泳分离DNA、聚丙烯酰胺凝胶电泳分离蛋白质，依据带电粒子在电场中迁移速率的差异实现分离。DNA因其磷酸骨架带负电，在电泳时向正极迁移^［24］^。

核酸在电场中迁移时，受两种方向相反的作用力^［25］^，即电场力（*F*）和摩擦力（*F*_f_），分别见式（1）和式（2）。


*F=qE=qU/d* （1）

*F*_f_
*=fv* （2）

其中，*q*为颗粒所带的电量；*E=U*/*d*为电场强度；*U*为两电极间的电势差（V）；*d*为两电极间的距离（cm）；*f*为摩擦系数（与颗粒的形状、大小和介质黏度有关）；*v*为核酸泳动速度（cm/s）；当颗粒以恒定速率移动时，*F=F*_f_，即*qE*=*fv*。在浓度一定的琼脂糖凝胶中，电泳迁移率（*μ*）可由式（3）计算得到。


*μ=v/E=q/f* （3）

由式3可知：核酸相对分子质量和形状不同，*μ*亦不同。相对分子质量越大，*f*越大，迁移速率越慢；相对分子质量相同时，线性DNA的*f*小于环状DNA，迁移速率更大。

### 1.2 信息编码原理

如图1所示，将M13mp18 DNA线性单链、149条短寡核苷酸链及5条检测链D_0_、D_1_、D_2_、D_3_和D_4_混合，在聚合酶链反应核酸扩增仪（PCR仪）中经“退火”程序形成线性DNA纳米开关。本实验中，检测链D_1_、D_2_、D_3_和D_4_以间隔600 nt的方式依次插入到线性DNA纳米开关中，构成一个4-bit的DNA纳米开关体系。未添加RNA保护链R_0_时，加入目标链T *_x_* 可同时与检测链D_0_及相应的D *_x_* 通过碱基互补配对，驱动开关发生从线性结构到环状的拓扑结构转变。具体而言，目标链T_1_、T_2_、T_3_、T_4_分别桥接D_0_与D_1_、D_0_与D_2_、D_0_与D_3_、D_0_与D_4_，从而诱导形成4种环状DNA纳米开关Loop 1~4。这些环状结构的相对分子质量相近，但环直径的大小依次递增（Loop 1<Loop 2<Loop 3<Loop 4），构成了一系列构型不同、可通过凝胶电泳区分的环状DNA纳米开关。不同DNA纳米开关在凝胶电泳中呈现差异化：开关Loop环直径越大，*f*越大，则*μ*越小。因此，该特性可用于二进制编码及基于凝胶电泳的信息可视化读取。

**图1:**
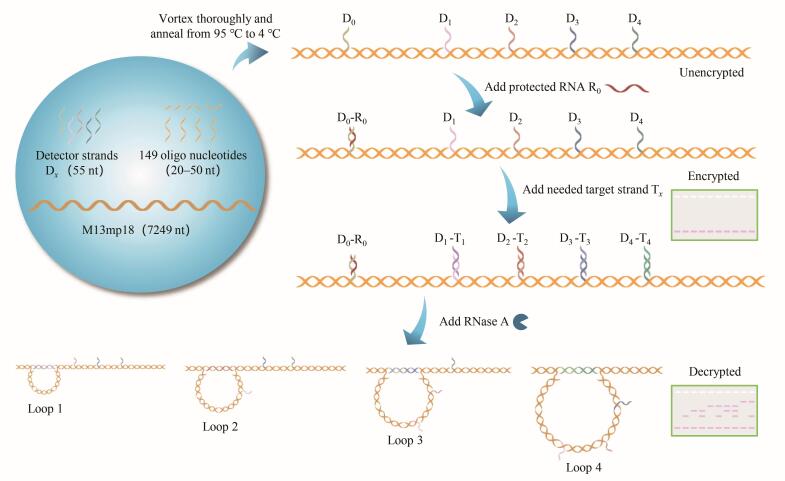
RNA保护调控的DNA纳米开关二进制加密原理图

本实验用DNA纳米开关在电泳中是否存在条带分别表示二进制的1和0，通过4种不同分子直径的DNA纳米开关组合对应4位的二进制编码（例如“1001”表示数字9）。如图2a所示，在凝胶电泳中，同一个泳道自上而下的条带分别对应Loop 4、Loop 3、Loop 2和Loop 1，依序表示2^3^、2^2^、2^1^和2^0^。通过识别条带存在（记为1）或缺失（记为0），并计算其对应二进制位的加权和（例如：“1001”表示2^3^+0+0+2^0^=9），可实现数字0~9的二进制编码。将编码不同数字的DNA纳米开关混合液按需组合，即可实现信息的分子存储。基于琼脂糖凝胶电泳，可对该存储信息进行可视化读取。

**图2:**
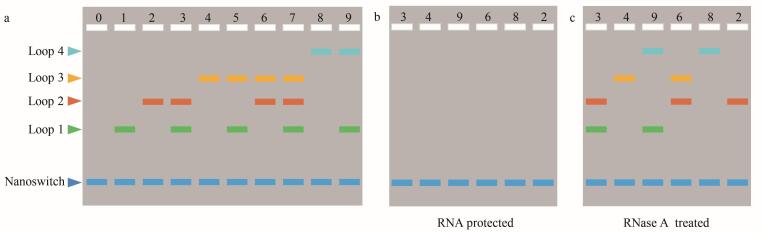
数字加密与解密示意图

### 1.3 信息加密-解密

根据有机化学“保护-脱保护”思想，本实验提出了RNA保护链调控机制，基于DNA纳米开关实现了信息的加密与解密。加密阶段，保护链R_0_与线性DNA纳米开关的检测位点D_0_碱基互补配对（图1），阻止目标链T *_x_* 与D_0_配对触发拓扑构象转换，此时凝胶电泳仅显示线性条带（图2b），无法读取存储信息，实现信息加密。解密阶段，利用RNase A酶对RNA的特异性降解作用，降解保护链R_0_后重新暴露出D_0_（图1）。此时，目标链T *_x_* 可触发纳米开关拓扑构象转换，形成环状DNA纳米开关。最后，通过琼脂糖凝胶电泳（图2c）即可实现信息的可视化读取。

本实验的教学重点在于：（1）理解基于RNA保护的DNA纳米开关的信息加密机制；（2）掌握琼脂糖凝胶电泳的原理和操作；（3）掌握基于酶降解实现信息解密的策略及操作。教学难点在于：如何利用RNA和RNase A酶对DNA纳米开关编码的信息进行充分保护及降解。在实验过程中，样品的配制、移液器的使用熟练程度、加样是否准确和在琼脂糖凝胶中的上样操作等因素均会影响学生实验的成败。

## 2 实验部分

### 2.1 仪器、试剂与材料

T100PCR仪和PowerPac^TM^ Basic琼脂糖凝胶电泳系统（美国Bio-Rad公司）；G：BOX Chemi XRQ化学发光成像系统（英国Syngene公司）。

环状M13mp18 DNA单链（陕西维世诺生物科技有限公司）；寡核苷酸序列短链（共149条，20~50 nt）、RNA保护链R_0_（1条，12 nt）、目标链D *_x_* （5条DNA单链，每条55 nt）和Na_2_EDTA∙2H_2_O（上海生工生物工程股份有限公司）；10倍浓度的Tris-硼酸缓冲液（10×TBE，上海生工生物工程股份有限公司）；0.5倍浓度的Tris-硼酸缓冲液（0.5×TBE，用10×TBE稀释得到）；10×Cut Smart buffer、RNase A酶、RNase H酶、脱氧核糖核酸酶I（deoxyribonuclease I，DNase I）酶和蛋白酶K（proteinase K）（New England Biolabs）；琼脂糖和氯化镁（分析纯，上海麦克林生化科技股份有限公司）；6×DNA上样缓冲液（6×Loading buffer）、10×Gel Red核酸染料（10 000×Gel Red稀释）、10×磷酸盐缓冲溶液（10×PBS）、1×磷酸盐缓冲溶液（1×PBS）（上海泰坦科技股份有限公司）。

### 2.2 表征方法

1.2%琼脂糖凝胶电泳：称取0.600 0 g琼脂糖，置于250 mL锥形瓶中，加入50 mL 0.5×TBE，摇匀后置于微波炉中加热得到凝胶溶液。冷却至60 ℃，插上梳子，倒入凝胶盒中，排尽气泡，静置20~30 min至凝胶凝固。加入少许0.5×TBE，轻轻拔出梳子，将凝胶放入电泳槽（注意：DNA带负电，从负极向正极迁移，样品孔一端应靠近负极）。在样品中，分别加入2 μL 6×Loading buffer和1 μL 10×Gel Red，混匀后依次上样。在室温下，以100 V电压（恒压）电泳60 min，结束后将凝胶放入凝胶成像系统进行成像和分析。

### 2.3 DNA纳米开关的制备

教师提前准备：（1）将149条100 μmol/L的寡核苷酸链各取1 μL混合于200 μL PCR管1中，同时将检测链D_0_~D_4_各取1 μL混合于200 μL PCR管2中。随后从管1和管2分别移取29.8 μL和1 μL至管3中混匀，配制混合物（Mixture）。（2）将环状M13mp18 DNA单链酶切成线性M13mp18单链备用，具体方法见参考文献［^21^］。

学生取PCR管5个，依次标记为A、B、C、D、E，分别在管中加入5 μL 100 μmol/L教师提前准备的线性M13mp18 DNA单链、0.8 μL教师提前准备的Mixture、1 μL 100 mmol/L MgCl_2_和3.2 μL超纯水。混匀后置于PCR仪中，先在95 ℃保持5 min，然后以1 ℃/min的速率降温至25 ℃。取出后加入190 μL 1×PBS缓冲液将产物稀释40倍，得到DNA线性纳米开关1 000 μL，确保后续实验用量。

另取5个PCR管，依次标记为Loop 1、Loop 2、Loop 3、Loop 4和Loops。向每管中依次加入稀释后的线性DNA纳米开关5 μL、10×PBS 1 μL和MgCl_2_溶液1 μL。随后，向管Loop 1中加入1 μL的目标链T_1_；管Loop 2中加入1 μL的目标链T_2_；管Loop 3中加入1 μL的目标链T_3_；管Loop 4中加入1 μL的目标链T_4_；在管Loops中加入目标链T_1_~T_4_各1 μL（共4 μL）。最后，向各管中加超纯水2 μL，使反应体系总体积为10 μL，混匀。将所有样品在室温下孵育30 min，以制备Loop环直径不同的环状DNA纳米开关Loop 1、Loop 2、Loop 3、Loop 4及管Loops中的Loop 1~4的混合物。最后，通过1.2%琼脂糖凝胶电泳分析环状M13mp18单链、线性M13mp18单链、标号为A、B、C、D、E的5个PCR管中的线性开关、环状DNA纳米开关Loop 1、Loop 2、Loop 3、Loop 4和Loop 1~4的混合物。

### 2.4 RNA调控机理验证

利用R_0_保护检测链D_0_，在反应体系中分别加入T_1_、T_2_、T_3_、T_4_和T_1_~T_4_的混合物，验证R_0_（50 µmol/L）对D_0_的保护效率。取12个PCR管，按表1配制反应体系。第1步在25 ℃孵育30 min保护D_0_；第2步加入目标链T *_x_* 在25 ℃孵育30 min实现信息加密；第3步在37 ℃反应10 min，使酶RNase A充分降解R_0_，实现对D_0_的脱保护；第4步在25 ℃孵育30 min使T *_x_* 与D_0_充分配对，实现解密。最后，通过1.2% 琼脂糖凝胶电泳进行信息可视化读取。

**表1:** RNA调控机理验证实验方案

Step	Added	PCR tubes											
		1	2	3	4	5	6	7	8	9	10	11	12
Step 1	diluted nanoswitch/μL	10	10	10	10	10	10	10	10	10	10	40	40
	R_0_/μL		1	1	1	1	1	1	1	1	1	4	4
	10×PBS/μL	2	2	2	2	2	2	2	2	2	2	8	8
	MgCl_2_/μL	2	2	2	2	2	2	2	2	2	2	8	8
	ultrapure water/μL	6	5	5	5	5	5	5	5	5	5	20	20
	Total/μL	20	20	20	20	20	20	20	20	20	20	80	80
Step 2	T_1_/μL			1	1							1	1
	T_2_/μL					1	1					1	1
	T_3_/μL							1	1			1	1
	T_4_/μL									1	1	1	1
Step 3	RNase A/μL				1		1		1		1		4
Step 4	incubate at 25 ℃ for 30 min to enable complete hybridization of T *_x_* and D_0_												

### 2.5 非特异性酶攻击测试

为评估加密系统对未授权解密尝试的抵抗力，本实验设计了非特异性酶攻击测试方案（表2）。通过比较经RNase A（授权解密酶）与其他3类未授权酶处理的解密效率，验证系统的特异性与抗干扰能力：①无酶处理（阴性对照）；②RNase A（阳性对照，10 mg/mL，37 ℃，10 min）；③RNase H（5 U/μL，37 ℃，30 min）；④DNase I（消化单/双链DNA，1 U/μL，25 ℃，30 min）；⑤蛋白酶K（20 mg/mL，55 ℃，60 min）。将含有纳米开关Loop 1的加密体系（R_0_保护+目标链T_1_）分别与上述酶1 μL在酶工作条件下共孵育。结束后统一在25 ℃孵育30 min使T *_x_* 与D_0_充分配对。最后，通过1.2%的琼脂糖凝胶电泳分析解密情况。

**表2:** 非特异性酶攻击测试实验方案

Step	Added	PCR tubes				
		1	2	3	4	5
Step 1	diluted nanoswitch/µL	10	10	10	10	10
	R_0_/µL	1	1	1	1	1
	10× PBS/µL	2	2	2	2	2
	MgCl_2_/µL	2	2	2	2	2
	ultrapure water/µL	5	5	5	5	5
Step 2	T_1_/µL	1	1	1	1	1
Step 3	RNase A/µL		1			
	RNase H/µL			1		
	DNase I/µL				1	
	Proteinase K/µL					1
Step 4	incubate at 25 ℃ for 30 min to enable complete hybridization of T *_x_* and D_0_					

### 2.6 信息“加密-解密”实验

由老师为每名学生随机指定1个6位数字密码，学生自行判断需要加入的目标链T *_x_* 进行实验。在实际教学中我们发现，很多同学无法准确判断需要加入的目标链。为了提高实验成功率，老师指导学生利用DeepSeek（DeepSeek-V3.1）开发了一款HTML程序，用于验证目标链选择是否正确，提高实验效率。第1步，每人取6个PCR管，按表2 Step 1相同比例配制RNA保护样品20 μL，25 ℃孵育30 min。第2步，经HTML程序核对无误后，在PCR管中分别加入所需目标链T *_x_* ，25 ℃孵育30 min。然后从每管移取10 μL至另取的6个PCR管中作为解密组，原来的6个PCR管作为加密组。第3步，在解密组中各加入1 μL RNase A酶，37 ℃孵育10 min使R_0_保护链被充分降解。第4步，加密组和解密组均在25 ℃孵育30 min，使解密组的目标链T *_x_* 与D_0_、D *_x_* 充分反应。最后，以1.2% 琼脂糖凝胶电泳对加密组与解密组的12个样品进行分析，实现密码信息的可视化读取。

## 3 结果与讨论

### 3.1 DNA纳米开关的构象表征

实验结果如图3所示：相比于线性M13mp18单链，制备的5份线性DNA纳米开关的迁移距离更短。说明形成线性开关后相对分子质量增大，迁移速率变慢（图3a）。从图3b可以观察到Loop 1~4的迁移距离随Loop环直径的增大而减小，说明环状DNA纳米开关制备成功。当加入所有T *_x_* 时，图3b泳道5中呈现出5个条带，自上而下依次对应Loop 4、Loop 3、Loop 2、Loop 1和nanoswitch。进一步证实了相对分子质量接近但构型不同的环状DNA纳米开关在同一浓度的凝胶中迁移速率不同。

**图3:**
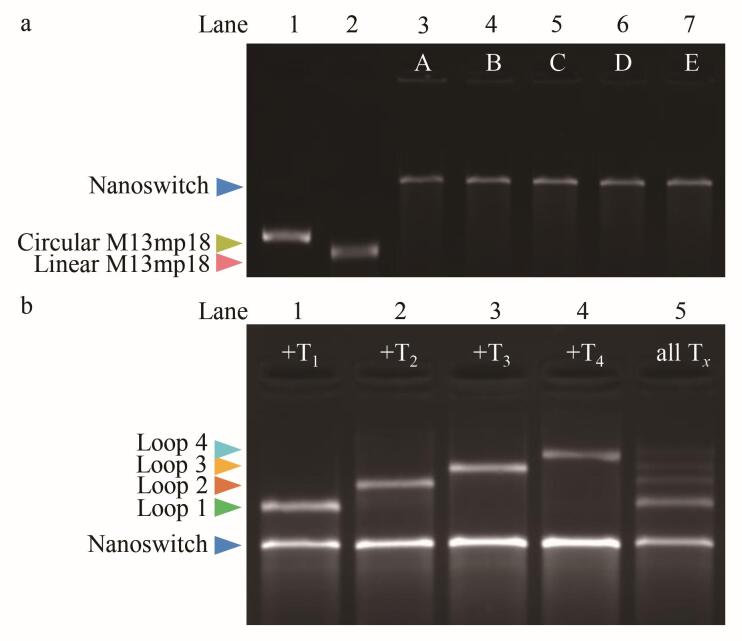
DNA纳米开关制备及验证

### 3.2 RNA保护链的加密效率与RNase A酶解密验证

分别将2.4节制备的样品用1.2%的琼脂糖凝胶进行分析表征，结果如图4所示。对比泳道1~3的条带可见：泳道3中没有观察到环状DNA纳米开关对应的条带，说明加入1 µL R_0_保护链（终浓度2.5 µmol/L）完全阻止了Loop 1的形成；泳道5、7、9的结果进一步说明，加入R_0_保护链后，其他目标链也无法触发开关从线性向环状结构转变。由此可见，R_0_的加入对所有存储信息实施了高效加密，泳道11的结果也支持该结论。随后，在加密体系中各加入1 µL RNase A进行酶切处理，泳道4、6、8、10、12中均出现了对应的Loop条带。这说明RNase A通过酶切有效降解了保护链R_0_，重新暴露了D_0_上的配对位点，使其得以与T *_x_* 结合，触发开关的拓扑构象转化，从而实现了信息解密。

**图4:**
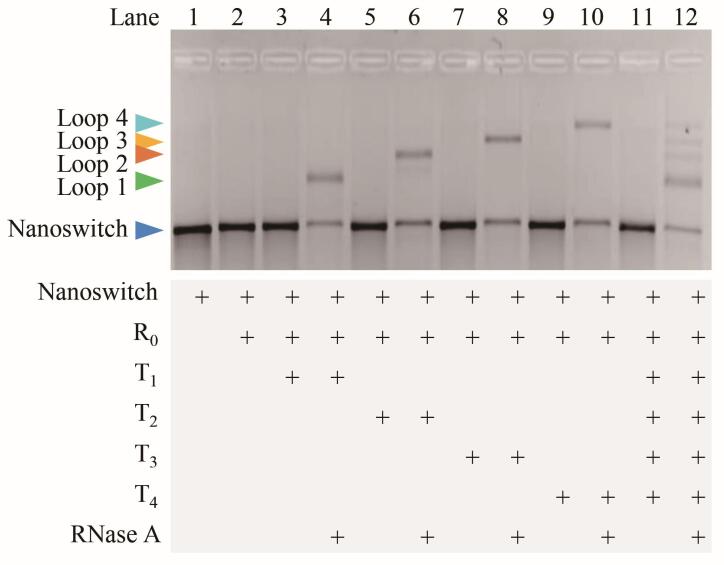
RNA调控机理验证

### 3.3 加密系统的特异性与抗干扰能力分析

对R_0_保护的纳米开关Loop 1进行酶攻击实验凝胶电泳结果如图5所示。以RNase A处理组为阳性对照，无酶处理组为阴性对照（Control）。电泳结果表明：泳道2（添加RNase A）：出现了Loop 1特征条带，表明RNase A降解了保护链R_0_，然后T_1_触发构象转换形成了Loop 1。泳道3~5（分别添加 RNase H、DNase I、蛋白酶K）：与泳道1（Control）一样均没有观察到Loop 1对应的条带（DNase I完全降解了DNA，泳道4中无条带显示）。说明除RNase A外，其他酶无法解除R_0_对D_0_的保护，本系统只能通过RNase A的特异性酶切才能实现可控解密。

**图 5:**
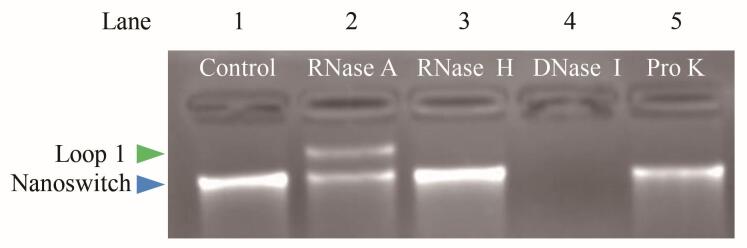
非特异性酶攻击测试凝胶结果图

### 3.4 信息“加密-解密”的可行性验证

学生独立开展加密-解密实验的结果如图6所示。加密阶段：3组样品经25 ℃孵育30 min后进行琼脂糖凝胶电泳分析，发现泳道中均仅显示线性DNA纳米开关对应的条带（图6中间图）。表明R_0_的存在成功阻断了目标链T *_x_* 与D_0_和T *_x_* 的碱基互补配对，实现了信息加密。解密阶段：加入RNase A进行特异性酶切后，琼脂糖凝胶电泳图（图6右图）呈现的条带与图6左图预设的信息完全吻合。说明本实验具有优异的成功率、可重复性和生物特异性解密等特征。

**图6:**
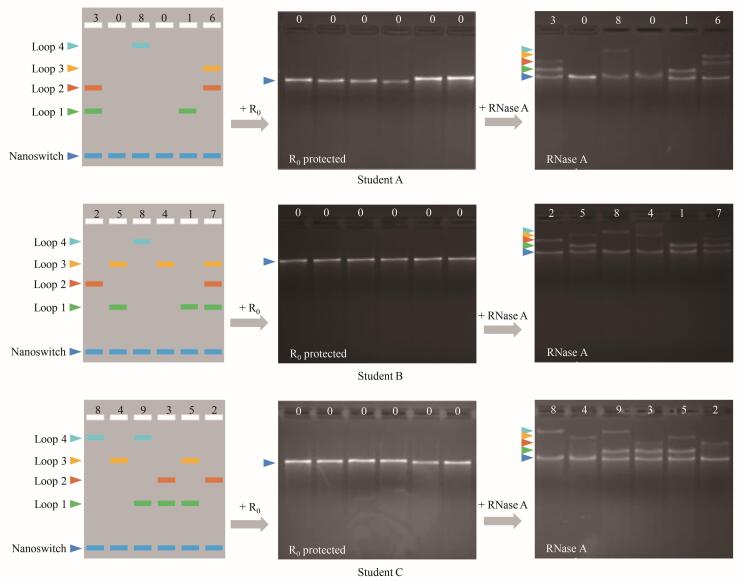
学生A、B、C信息加密-解密结果示意图

## 4 实验的组织实施及教学反思

### 4.1 实验的组织实施

本综合实验采取模块化教学，总用时不超过12 h，分为DNA纳米开关制备、RNA调控机理验证、非特异性酶攻击实验、信息“加密-解密”实验4个模块。通过琼脂糖凝胶电泳将4个模块串联成一个综合实验。每个模块都会开展“制胶-上样-跑胶-成像分析”的练习，通过4次重复练习帮助学生充分掌握凝胶电泳的原理及操作。

其中，模块1“DNA纳米开关制备”用时约170 min，学生需制备Loop环直径不同的环状DNA纳米开关并通过琼脂糖凝胶电泳进行表征。模块2“RNA调控机理验证”用时约170 min，学生需使用R_0_对检测链D_0_进行保护、使用酶RNase A充分降解R_0_实现对D_0_的脱保护并进行凝胶电泳的验证。模块3“非特异性酶攻击测试”用时约220 min，学生需要通过不同酶对加密体系进行脱保护实验，并通过琼脂糖凝胶电泳分析解密情况。模块4“信息‘加密-解密’实验”用时约160 min，学生需根据实验原理完成老师随机分发的密码的加密和解密实验，并通过琼脂糖凝胶电泳进行验证。当然，可以根据教学具体情况，灵活安排实验内容，如在教学过程中同步进行多个模块的实验，可以大幅缩短实验所需时间。

### 4.2 教学反思

本实验综合运用化学保护策略、二进制算法与DNA纳米技术，将琼脂糖凝胶电泳技术贯穿到整个实验中，实现了从单一的DNA分离向分子构效关系解析的拓展。通过跨学科思维训练与前沿技术实践，旨在引导学生掌握琼脂糖凝胶电泳与成像分析、分子信息编码等基础理论与操作技能，提升其跨学科整合与解决实际问题的能力。因此，该实验可作为创新性综合实验或选修实验，面向化学类、医药类、环境类等专业二年级及以上本科生开设。

在教学实施过程中，将琼脂糖凝胶电泳技术贯穿整个实验流程。通过模块化设计及4次重复训练，有效强化了学生的操作能力，并深化了他们对技术原理的理解。从实施情况来看，绝大多数实验小组表现出浓厚的兴趣，并能在12 h内完成实验。尤为可喜的是，部分学生展现了出色的创新能力，如提出通过保护不同检测链实现信息加密与解密，或利用该策略实现二进制数的四则运算，充分体现了其创新思维与跨学科思维能力的提升。

然而，教学过程中也发现部分学生的实验结果与预期存在偏差，主要体现在以下3个方面：（1）对实验原理理解不足，导致目标链选择错误。为此，教师指导学生利用DeepSeek平台开发了HTML密码验证程序，通过模拟验证增强理解，提高实验成功率。（2）微量移液器操作不熟练，影响加样精度。实验前安排了专项练习，要求学生移取1 µL超纯水并用分析天平称量，以量化评估操作准确性。（3）凝胶上样时因样品孔可视性差，易戳破胶孔或加样不全。通过在电泳槽下方垫白纸增强对比度，有效改善了上样准确性。

此外，为确保实验安全，所有学生在实验前须完成安全培训。培训内容需强调：处理高温琼脂糖溶液时应穿戴耐热手套及护目镜；连接电泳槽电极时须确保正负极正确对应；含有核酸染料（如 GelRed^TM^）的凝胶需经高压蒸汽灭菌后，再置于生物危害专用容器中废弃处理；实验全程应佩戴丁腈手套，避免直接接触潜在生物或化学风险物质。

目前，本实验已在我校环境类、材料类和资源类专业中完成了初步教学实践。随机调查显示，绝大多数学生认为该实验融合了多学科知识，能够将理论知识与实际问题紧密结合，具有较强的挑战性，激发了大家的科研兴趣。在教学过程中，师生围绕实验改进和深度研究方案展开了积极互动，实现了教学相长，整体教学效果令人满意。

## 5 结语

全球数据的指数级增长对新型存储策略的开发提出了迫切需求，并推动了多种新策略的探索^［26］^。其中，蛋白质^［27，28］^和DNA^［29］^等生物大分子作为天然信息载体，被视为极具潜力的下一代信息存储介质。而DNA用于数据存储优势明显，包括存储密度高，存储时间长，维护能耗低等^［30］^。与此同时，DNA纳米技术的快速发展不断推动数据存储及读取技术不断进步，如基于荧光探针、光电信号和凝胶电泳^［23］^等方法的高效信息读取技术已取得了长足发展。

在此背景下，开发基于DNA纳米开关的二进制信息加密和解密实验，不仅有助于学生了解学科前沿动态，更能引导他们将理论知识与实际应用相结合，运用先进生物技术解决具体问题。教学实践表明：学生均可独立完成加密-解密操作（耗时约12 h），并准确解析凝胶电泳图谱中蕴含的二进制信息。本实验兼具创新性与教学适配性：将DNA纳米加密成果转化为可重复的本科教学实验，不仅帮助学生掌握了琼脂糖凝胶电泳的原理和技能，而且深化了学生对电泳分离机制与生物分子构效关系的理解。此外，本实验具有较强的可复制性，易于在其他高校本科实验教学中进行推广。
